# Efficacy and Safety of Repeated Percutaneous Radiofrequency Thermocoagulation for Recurrent Trigeminal Neuralgia

**DOI:** 10.3389/fneur.2018.01189

**Published:** 2019-01-18

**Authors:** Guangzhao Liu, Yumin Du, Xiaowen Wang, YuE Ren

**Affiliations:** Department of Pain Management, The Second Hospital of Hebei Medical University, Shijiazhuang, China

**Keywords:** trigeminal neuralgia, radiofrequency thermocogulation, trigeminal ganglion, trigeminal nerve, pain

## Abstract

**Background:** Percutaneous radiofrequency thermocoagulation (PRT) is used to treat trigeminal neuralgia (TN) with a satisfactory pain relief but a high recurrence rate.

**Objective:** To explore the efficacy and safety of repeated PRT for recurrent TN as compared to patients who received the first PRT.

**Methods:** Between January 2013 to May 2013, 31 patients with recurrent TN who have been treated with PRT previously were recruited and underwent repeated PRT (group A), and compared with 41 TN patients received the first initial PRT (group B). Visual Analog Scale (VAS) score was assessed preoperatively and postoperatively after 2 years of follow-up, and compared in terms of initial pain relief, complications, and recurrence rate between the two groups.

**Results:** In group A, 27 patients (87.0%) were pain free immediately, and 30 patients (96.8%) experienced pain relief at 48 h, whereas that was 37 patients (90.0%) and 40 patients (97.6%) in group B (*p* ≧ 0.05). Patients in group A who remained an “excellent” or “good” pain relief condition (VAS score ≦ 1) were 96.8% at 6 months, 83.9% at 1 year, 74.2% at 2 years, whereas the percentage in group B was 97.6, 85.4, and 73.2% (*p* ≧ 0.05).

**Conclusion:** For patients with recurrent TN after PRT, repeated PRT might be considered as a useful treatment option when other treatments fail. In addition, the frequency and severity of adverse events for repeated PRT were similar as compared to initial PRT.

## Introduction

Trigeminal neuralgia (TN), also called as “tic douloureux,” is a sudden, usually unilateral, severe, brief, stabbing, and recurrent pain in the distribution of the trigeminal nerve with a prevalence of 4 per 100,000 in the general population (1). Many treatment options have been developed so far to afford pain relief in patients with TN. Up to date, drug therapy, percutaneous radiofrequency thermocoagulation (PRT) of trigeminal ganglion (2), glycerol injection, balloon compression, stereotactic radiosurgery, and microvascular decompression (MVD) have been used to treat TN. Among them, the first-line therapy is pharmacological therapy as it is immediately available and usually effective in most cases. When pharmacological therapy fails due to either poor pain control or intolerable side effects, other techniques such as PRT and MVD are often considered.

A number of studies have been done to compare the efficacy and safety of different techniques (3–6). According to the most recent publications, MVD offers the best results in terms of improvement of quality of life and pain relief over the long term (7). PRT offers a good efficacy (over 90%), but with a high relapse rate (15–20%) (8–10). However, it should be noted that PRT is usually a better choice for the elderly patients due to its lower morbidity and mortality as compared to MVD (11). In addition, PRT offers the highest rate of complete pain relief (12).

PRT can be undergone in the same patient when TN relapses, especially for patients at an older age (13). Some previous studies have investigated whether repeated PRT can be used in patients with recurrent TN (13–16), but most of them did not compare the efficacy and safety of repeated PRT with the control group, i.e., patients treated with initial PRT. Such knowledge is highly needed as recurrent TN is a big challenge to clinical doctors and it is still unclear whether repeated PRT can be effective for patients with recurrent TN, especially for those who cannot undergo MVD therapy because of its associated morbidity or mortality. In addition, the frequency and severity of adverse events for repeated PRT were largely unclear, which we aimed to explore in this study.

## Methods

### Patients

This study was approved by the Ethical Committee of the Second Hospital of Hebei Medical University, and the written informed consent (including image for publication) was obtained from all the patients prior to involvement in the study. All methods were performed in accordance with the relevant guidelines and regulations of the Hospital. From January 2013 to May 2013, a total of 72 patients with TN were underwent 3-D CT-guided PRT at the Hospital and were enrolled in our study. Among them, 31 patients who have received PRT before were treated with repeated PRT (group A), and 41 TN patients who never received any operation were treated with initial PRT (group B). All of these patients were followed for 2 years.

All the patients were selected based on the following criteria: (1) primary TN of maxillary and/or mandibular divisions over one year based on symptoms described by the International Headache Society Classification, (2) failure of pharmacological management by multiple antiepileptic drugs or recurrence after PRT, (3) a pain visual analog scale (VAS) score over 7/10 and with a poor quality of life because of the pain, (4) any one or more of the following: poor candidacy for general anesthesia, suboccipital craniotomy, patient preference to avoid craniotomy for MVD, and (5) normal brain magnetic resonance imaging.

Patients who had undergone other invasive or noninvasive management (balloon compression, glycerol rhizotomy, gamma knife, MVD) and patients with facial pain other than TN were excluded from the study.

### PRT Operation

A detailed medical history was ascertained and routine blood investigations were done preoperatively in all patients. CT/MRI imaging of the head was also examined.

The operation was done in the CT operation theater with sterilization. The operation procedure was explained to the patients before the operation, and it was performed under 3-D CT guidance. Patients were fasted for at least 6 h before the operation and prophylactic antibiotic was administered 1 h before the operation. Electrocardiogram, blood pressure and pulse oximetry were monitored during the operation. Low dose of intravenous fentanyl and midazolam were usually administered for analgesia and sedation. The patients were awake during the operation.

All patients underwent PRT (initial or repeated) by a single surgeon to avoid heterogeneity of operating bias. According to the Sweet technique, the patient lied in the supine position on the table, and the head and neck were in a normal position. Hartel's markings were drawn over the face with marking pencil, and the point of entry of the needle was usually 3 cm to the angle of mouth. Two other points were (1) medial margin of pupil and (2) a point 2.5 cm anterior to tragus on the zygomatic arch to serve as planes for diverting needle to foramen ovale (17).

**Figure 1:**
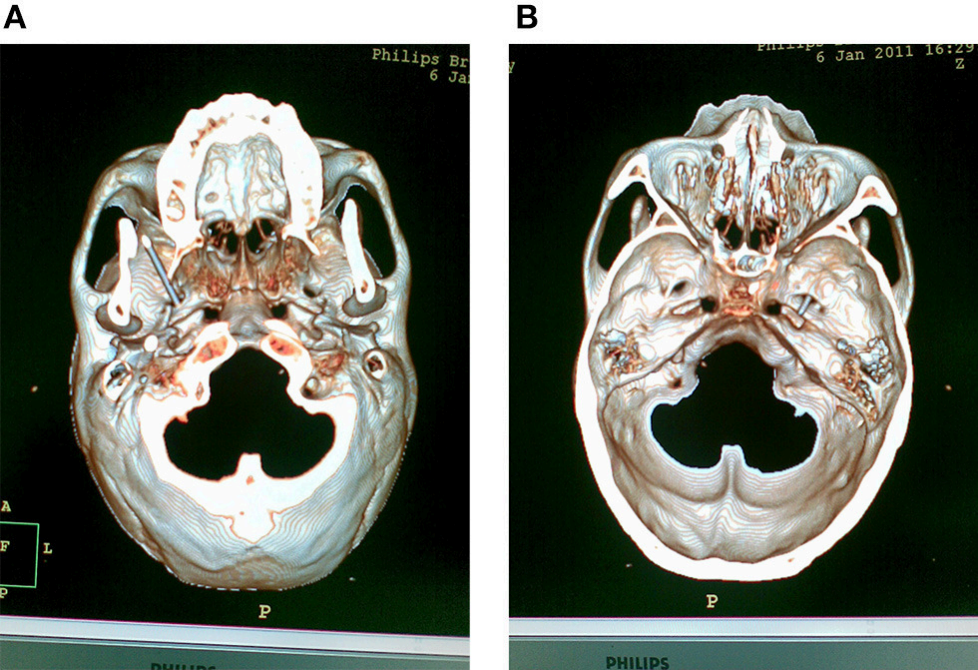
CT scans were performed to confirm the correct localization and the depth of the cannula tip into the foramen ovale. **(A)** The 3-D scaphion view image showed the localization of the cannula in the foramen ovale. **(B)** The 3-D image of the medial view of skull base showed the depth of the cannula tip into the foramen ovale.

After local infiltration of 0.5% lidocaine (10 ml) along the trajectory of cannulation over the skin and subcutaneous tissue, a 22-gauge, 10-cm radiofrequency cannula with a 5-mm active tip was used. The cannula followed a straight line directed toward the pupil when seen from the front and passed 3 cm anterior to the external auditory meatus when seen from the side, and then set smoothly around the foramen ovale without patient discomfort. Once the needle reached the skull base, screening skull base CT was performed (at 2-mm intervals, 120 kV, 500 mAs, Philips Brilliance CT, Holland) to achieve the 3-D image and to confirm the relationship between the localization of needle tip and the foramen ovale. Based on the 3-D image, the needle was adjusted to enter the foramen ovale. T Entry into foramen ovale was easy performed in most of cases, but a few cases were difficult to reach and need a few tries. CT scans were performed to confirm the correct localization and the depth of the cannula tip into the foramen ovale (Figure 1).

For different trigeminal nerve rootlets afferent into trigeminal ganglion, each rootlet received sensory input from different facial regions. Stimulation at 50 Hz, 0.1–0.2 V was used to identify the nerve division to make sure the electrode tip reaching the position, while enlisting the patient's subjective report of sensation in response to stimulation. The stylet was then removed from the cannula, and aspiration was performed to ensure that there was no cerebrospinal fluid (CSF) or blood. If CSF was aspirated, the localization of the cannula tip would be adjusted. Injecting of 2% lidocaine (0.2 ml) into the trigeminal ganglion helped to alleviate pain of the thermocoagulation. PRT lesioning at 75°C was carried out for 90 s. To achieve effective pain relief, the cannula tip usually was repositioned, and additional thermal lesioning was performed. After this, the cannula was removed, and the patients were carried to the ward and discharged after 48 h. The operation was repeated if satisfactory pain relief was not achieved after 48 h.

### Efficacy

A visual analog scale (VAS), consisting of a 10 cm line with two endpoints labeled as “no pain” and “most intense pain imaginable,” was used to assess pain intensity. VAS scores were obtained preoperatively and 48 h postoperatively via face-to-face interviews. Final outcome was divided into three categories: excellent, good, and poor. “Excellent” was used for those patients who were pain free; “good” used for those who were in pain but with VAS score ≦2 without medications at 48 h; “poor” used for those who were in pain, with VAS score ≧3 with or without medications.

### Recurrence Rate

Recurrence was defined as a return of trigeminal pain, which had the same characteristics as pre-operatively, with VAS score ≧3, and pain frequency ≧3 times a day without medication use. All patients were followed and examined at 6 months, 1 and 2 years. VAS scores after 2 years of follow-up were obtained via telephone interviews by an independent doctor who was not involved in the study, and the recurrence time was also recorded.

### Complications During the Operation

Nausea, vomiting, heart rate change (bradycardiaor tachyarrhythmia), blood pressure change (hypertension or hypotension) and other complications (other cranial nerve paresis, cerebrospinal fluid fistula, and intracranial hemorrhage) during the operation were recorded. Regarding long-term outcomes, all the patients were interviewed about masticatory weakness, dysesthesia, and corneal numbness after PRT.

### Statistics

SPSS version 13 (SPSS Inc. Chicago, IL, USA) was used for statistical analysis. Data were analyzed in terms of operation efficacy, recurrence of pain, and complications. Nonparametric data were analyzed using the Mann Whitney test. Other data were analyzed using the Student's *t*-test between the two groups. Categorical data were analyzed using the Fisher's exact test. The results were presented as mean (SD). Differences were considered statistically significant when *P* < 0.05.

## Results

From January 2013 to May 2013, a total of 72 patients with TN were underwent 3-D CT-guided PRT at The Second Hospital of Hebei Medical University and were enrolled in our study. Among them, 31 patients with recurrent TN were treated with repeated PRT (group A), and 41 TN patients were treated with initial PRT (group B).The baseline characteristics of the patients are summarized in Table 1. Female outnumbered male in both groups. The mean ± SD age was 68.52 ± 11.61 years in group A and 67.15 ± 11.57 years in group B, respectively. Most of these patients were right-side involved with 61.3% in group A and 61.0% in group B. Mandibular nerve was involved in 58.1% in group A and in 48.8% in group B, whereas both maxillary and mandibular nerves were involved in 41.9% in group A, and 51.2% in group B. The mean ± SD duration of neuralgia was 9.94 ± 5.69 years and 10.12 ± 5.73 years in the group A and the group B, respectively. Facial hypoesthesia existed in 10 (32.3%) patients in the group A, and none of them was noted in the group B preoperatively. All of the 10 patients felt mild facial hypoesthesia.

**Table 1:** Comparison of demographic and clinical characteristics at the baseline between group A and group B.

	**Group A, *n* = 31**	**Group B, *n* = 41**	***P*-value**
Age, y (Mean ± SD)	68.52 ± 11.61	67.15 ± 11.57	0.621
Gender, no. (%)			0.814
Male	12 (38.7)	17 (41.5)	
Female	19 (61.3)	24 (58.5)	
Duration of neuralgia, y	9.94 ± 5.69	10.12 ± 5.73	0.891
Branch affected, no. (%)			0.435
V2+V3	13 (41.9)	21 (51.2)	
V3	18 (58.1)	20 (48.8)	
Side affected, no. (%)			0.978
Left	12 (38.7%)	16 (39.0%)	
Right	19 (61.3%)	25 (61.0%)	
Facial hypoesthesia, no. (%)			
Mild	10 (32.3%)	0 (0%)	0.000
Moderate	0 (0%)	0 (0%)	
Severe	0 (0%)	0 (0%)	

*Data are presented as number of patients (%) or Mean ± SD. SD, standard deviation; V2, maxillary division; V3, mandibular division*.

### Efficacy

There was no technical failure in our study, and a successful entry into foramen ovale was achieved in all cases. After repeated PRT, the rate of complete pain relief (excellent) was 96.8% (30/31) in the group A, whereas it was 97.6% (40/41) among patients received initial PRT in group B. One patient in the group A and one in the group B required a repeated operation.

### Complications During the Operation

The complications during the operation were recorded (Table 2), and there was no difference between the two groups. Facial hypoesthesia occurred in all patients (100% in group A, and 100% in group B) immediately after operation. All of them felt moderate facial hypoesthesia postoperatively. Nausea and vomiting occurred in 4 (12.9%) patients in the group A, and 5 (12.2%) in the group B during the operation. Heart rate change (bradycardia or tachyarrhythmia) occurred in 22 (71.0%) patients in the group A, and 30 (73.2%) in the group B during the operation. Hypertension occurred in 20 (64.5%) patients in the group A, and 29 (70.7%) in the group B. No hypotension was noted.

**Table 2:** Comparison of complications during the operation between group A and group B.

	**Group A, *n* = 31**	**Group B, *n* = 41**	***P*-value**
Facial hypoesthesia	31 (100)	41 (100)	1.000
Nausea vomiting	4 (12.9)	5 (12.2)	0.787
Heart rate change	22 (71.0)	30 (73.2)	0.836
Blood pressure change	20 (64.5)	29 (70.7)	0.575
Masticatory weakness^a^	3 (9.7)	4 (9.8)	0.696

*Data are presented as number of patients (%)*.

a *Masticatory weakness 48 h after the operation*.

Masticatory muscle weakness occurred in 3 (9.7%) patients in the group A, and 4 (9.8%) patients in the group B. There was no corneal numbness, keratitis, other cranial nerve paresis, cerebrospinal fluid fistula, anesthesia dolorosa, and intracranial hemorrhage during and after PRT operations and no death was reported.

### Recurrence of TN

The percentage of recurrent TN at 6, 12, and 24 months was 3.2, 9.7, and 19.4% in the group A, whereas it was 2.4, 9.8, and 17% in the group B (Table 3). There was no significant difference.

**Table 3:** Comparison of recurrence of pain between group A and group B.

	**Group A, *n* = 31**	**Group B, *n* = 41**	***P*-value**
Recurrence of pain at 6 month	1 (3.2)	1 (2.4)	1
Recurrence of pain at 12 month	3 (9.7)	4 (9.8)	0.788
Recurrence of pain at 24 month	6 (19.4)	7 (17.1)	0.759

*Data are presented as number of patients (%)*.

## Discussion

TN is a disorder of the trigeminal nerve that results in intense episodic pain, which deteriorates the quality of life. A wide range of treatment strategies are available, and PRT has been shown to be a well-established treatment modality (18, 19). Percutaneous trigeminal lesioning for the treatment of trigeminal neuralgia was first descripted in 1914 by Hartel (20). White and Sweet refined the procedure with the use of a short-acting anesthetic agent, electrical stimulation (21, 22). With the aid of image-guided cannulation of the foramen ovale, such as CT and fluoroscopy, the procedure becomes much easier and safer in recent years. The rationale of PRT on TN is to interrupt the peripheral stimuli to reach the central nervous system (23). It can be performed in the same patient more than one time if required, but its efficacy and safety is still unclear, especially for patients in China, which we have tried to explore in this study.

CT imaging-guided localization modalities are by far the most superior strategy. The addition of three-dimensional (3-D) imaging reconstruction has produced more effective and safe results than two-dimensional imaging (24). Three-dimensional reconstruction can assist the surgeon to have a clear solid of the foramen ovale with different shape in each patient. In our study, anatomical variations in size and shape of foramen ovale could often be observed, including the integration of the foramen ovale and the foramina spinosum. Sometimes there is skull deficit at anterolateral aspect of the foramen ovale in some patients, which can result in catastrophic complications without precaution. Three-dimensional imaging reconstruction of the skull base should be obtained before the operation, which helps to find the anatomical variations and to avoid inadvertent injury to the surrounding neurovascular structures. The location into the foramen ovale and depth of penetration of the cannula are visual, which confers confidence on the surgeon and improves the safety of the patients immensely. In addition, for patients with a bony prominence around the foramen ovale which can interfere a successful cannulation during conventional technique, the three-dimensional reconstruction image is more effective and accurate than other techniques. As a general precaution, it is recommended that the needle should be directed at the anterolateral aspect of the foramen ovale and “walked” into the foramen (25). This decreases the possibility of proximity of the carotid artery and the middle meningeal artery. In addition, the patient can lay in the supine position on the table, with the head and neck in a usual position guided by three-dimensional imaging. Such techniques are more comfortable for rachiocyphosis patients and some old patients than those guided by two-dimensional imaging, which need neck extending and sometimes it is impossible for these patients.

According to a prospective study of 154 patients treated by PRT and were followed for 15 years (9, 25), 153 (99%) of them obtained initial pain relief after PRT and pain persisted in only one (1%) patient. Another study based on 1561 patients reported a rate of 97.6% of initial pain relief (12). In our study, we found that the rates of immediate pain relief and pain relief at 48 h were largely similar in patients treated with repeated PRT and those with initial PRT, suggesting that repeated PRT was a good option for patients with recurrent TN. In addition, the recurrent rate was comparable between the two groups after 2 years of follow-up, and they were consistent with some previous reports (8, 9). Our data suggested that the efficacy of repeated PRT was comparable to initial PRT.

The main problems preventing widespread use of PRT are the side effects. We further tested the safety of repeated PRT as compared to initial PRT. We found that there was no difference of complications during and after operation between the two groups, suggesting repeated PRT was a safe operation procedure for patients with recurrent TN. The observed complications in our study were largely consistent with previous reports. Masticatory weakness was reported to be as high as 29%, which was higher than the data in the present study. We found that masticatory muscle weakness occurred in around 10% patients in both groups, which may be due to (1) improved imaging and needle localization by 3-D CT and (2) accurate mapping before lesioning.

Recurrence rate after treatment is another important issue in the treatment for TN. Two studies reported that the recurrence rates ranged from 7.8 to 25% at 11.6 and 14 years, respectively (8, 9). The recurrence rate for patients received initial PRT was comparable to patients received repeated PRT in our study, suggesting that repeated PRT has a comparable recurrence rate as compared to initial PRT.

A few limitations need to be kept in mind when interpreting the data. First, the patients with V1 or V1+V2 TN were excluded. PRT of supraorbital nerve was performed for isolated 1st-division TN to preserve the corneal reflex, and PRT of supraorbital nerve and maxillary nerve for 1st and 2nd-division TN were done. Another limitation was that the size of the study sample was small and the duration of follow-up was relatively short. However, all of these patients will be continuously monitored, and further reports could give even clear picture of repeated PRT on recurrent TN.

## Conclusion

In general, repeated PRT is a safe and effective treatment for patients with recurrent TN. In addition, the frequency and severity of adverse events for repeated PRT were similar as compared to initial PRT.

## Author Contributions

All authors listed have made a substantial, direct and intellectual contribution to the work, and approved it for publication.

### Conflict of Interest Statement

The authors declare that the research was conducted in the absence of any commercial or financial relationships that could be construed as a potential conflict of interest.
